# Ultra-deep T cell receptor sequencing reveals the complexity and intratumour heterogeneity of T cell clones in renal cell carcinomas

**DOI:** 10.1002/path.4284

**Published:** 2013-11-12

**Authors:** Marco Gerlinger, Sergio A Quezada, Karl S Peggs, Andrew JS Furness, Rosalie Fisher, Teresa Marafioti, Vishvesh H Shende, Nicholas McGranahan, Andrew J Rowan, Steven Hazell, David Hamm, Harlan S Robins, Lisa Pickering, Martin Gore, David L Nicol, James Larkin, Charles Swanton

**Affiliations:** 1 Cancer Research UK, London Research InstituteUK; 2 Barts Cancer Institute, Barts and the London School of Medicine and DentistryLondon, UK; 3 UCL Cancer InstituteLondon, UK; 4 Royal Marsden HospitalLondon, UK; 5 Departments of Pathology and Histopathology, University College HospitalLondon, UK; 6 Centre for Mathematics and Physics in the Life Sciences and Experimental Biology (CoMPLEX), University College LondonUK; 7 Adaptive BiotechnologiesSeattle, WA, USA; 8 Fred Hutchinson Cancer Research CenterSeattle, WA, USA

**Keywords:** cancer immunity, biomarker, immunotherapy, T cell, intratumour heterogeneity

## Abstract

The recognition of cancer cells by T cells can impact upon prognosis and be exploited for immunotherapeutic approaches. This recognition depends on the specific interaction between antigens displayed on the surface of cancer cells and the T cell receptor (TCR), which is generated by somatic rearrangements of TCR *α*- and *β*-chains (TCRb). Our aim was to assess whether ultra-deep sequencing of the rearranged TCRb in DNA extracted from unfractionated clear cell renal cell carcinoma (ccRCC) samples can provide insights into the clonality and heterogeneity of intratumoural T cells in ccRCCs, a tumour type that can display extensive genetic intratumour heterogeneity (ITH). For this purpose, DNA was extracted from two to four tumour regions from each of four primary ccRCCs and was analysed by ultra-deep TCR sequencing. In parallel, tumour infiltration by CD4, CD8 and Foxp3 regulatory T cells was evaluated by immunohistochemistry and correlated with TCR-sequencing data. A polyclonal T cell repertoire with 367–16 289 (median 2394) unique TCRb sequences was identified per tumour region. The frequencies of the 100 most abundant T cell clones/tumour were poorly correlated between most regions (Pearson correlation coefficient, –0.218 to 0.465). 3–93% of these T cell clones were not detectable across all regions. Thus, the clonal composition of T cell populations can be heterogeneous across different regions of the same ccRCC. T cell ITH was higher in tumours pretreated with an mTOR inhibitor, which could suggest that therapy can influence adaptive tumour immunity. These data show that ultra-deep TCR-sequencing technology can be applied directly to DNA extracted from unfractionated tumour samples, allowing novel insights into the clonality of T cell populations in cancers. These were polyclonal and displayed ITH in ccRCC. TCRb sequencing may shed light on mechanisms of cancer immunity and the efficacy of immunotherapy approaches. Copyright © 2013 Pathological Society of Great Britain and Ireland.

## Introduction

T cells can infiltrate solid tumours and recognize peptide antigens presented on the surface of cancer cells by MHC molecules. Such antigen-specific adaptive T cell immune responses can be directed towards self antigens 1–4 as well as neo-antigens (or ‘mutated self-antigens’) generated by somatic mutations in the cancer genome 5,6. T cells directed towards cancer cells can significantly impact upon clinical outcome, as shown by a number of studies that have found a correlation between increased numbers of tumour-infiltrating lymphocytes and improved survival in a variety of tumour types 7. More recently, immunotherapeutic approaches based on antibody-mediated blockade of co-inhibitory receptors expressed on activated T cells (CTLA4 and PD-1) have been shown to produce durable responses, even in some patients with highly pretreated solid tumours, including ccRCC 8–10. Whilst a large number of studies have focused on understanding the mechanisms regulating T cell function within the tumour microenvironment, the clonal complexity of the T cell response in solid tumours throughout progression and therapy, and the potential utility of the T cell clonality patterns as a prognostic or predictive biomarker capable of informing choice of therapy, remain unexplored.

The repertoire of antigen-specific T cells is generated during T cell differentiation in the thymus, by somatic recombination of the T cell receptor *α*- and *β*-chains 11. Rearrangement of V, D and J segments in the TCRb generates the highly variable complementary determining region 3 (CDR3) critical to determine the specificity of each particular T cell clone. ImmunoSEQ-technology allows ultra-deep sequencing of the TCRb CDR3 region and reveals the clonal composition of T cell populations 12. This ultra-deep TCR-sequencing method has been applied to DNA extracted from purified immune cell samples to define the T cell repertoires in the blood of healthy individuals 13, during viral infections 14 or following treatment for leukaemia 15. More recently, this technology has been was used to interrogate T cell clonality directly from the DNA extracted from unfractionated cancer samples, which contain a mixture of cancer and stroma cells as well as lymphocytes 16. However, some tumour types, such as clear cell renal cell carcinomas (ccRCCs), can display extensive genetic intratumour heterogeneity 17,18 which, as seen for other cancers 19,20, is likely to generate neo-antigens with a heterogeneous distribution throughout the tumour. Our aim was to analyse multiple samples from individual ccRCCs with ultra-deep TCR-sequencing to determine whether T cell infiltrates were uniform or heterogeneous across different regions of the same tumour. Deciphering the heterogeneity of the T cell repertoire within individual tumours may have important implications for future biomarker discovery immunotherapy studies.

## Materials and methods

All patients had been diagnosed with stage IV renal ccRCCs. One of the included patients was treatment-naïve, one had been pretreated with sunitinib for 16 weeks and two further patients had been treated on the EPREDICT trial (EudraCT No. 2009-013381-54) with 6 weeks of pre-operative everolimus, followed by a nephrectomy. The translational research study presented here had been approved by an ethics review board and all patients had provided written informed consent for participation. Multiple tumour specimens were collected as described previously 17. In brief, the nephrectomy specimen was dissected by a uro-oncology pathologist and different regions, representing the spatial extent and macroscopic heterogeneity of each primary tumour, were harvested (each ca. 5 × 5 × 5 mm in size) and snap-frozen in liquid nitrogen within 1 h after surgical resection of the primary ccRCC. DNA was extracted and three or four samples/tumour with a good DNA yield were chosen for TCRb sequencing. 2 µg DNA, or lower amounts from tumour regions with restricted DNA availability (for details see Table 1), were sent to Adaptive Biotechnology (Seattle, USA). Adaptive Biotechnology performed the ’survey’ level ImmunoSEQ assay 12 on each sample. The resulting sequence data was processed and uploaded onto the ImmunoSEQ Analyser web-based relational database for analysis. Sequencing error correction, as previously described 12, was applied automatically by the analysis platform to support the precise quantification of rare T cell clones 21. Data were normalized for PCR bias as recommended by Adaptive Biotechnologies and regional TCRb sequences defining individual T cell clones and their regional abundance were downloaded from the webpage for the analyses specified. We used the Spearman’s rank test to assess the relationship between total TCRb reads and immunohistochemical T cell infiltrate across all regions from all tumours. A linear regression function was computed after exclusion of data from R6 RMH002, which was a single outlier in the dataset. All other correlations were analysed by the Pearson correlation coefficient method. The statistical significance of the difference in TCR reads between everolimus-treated and untreated samples was determined by a two-sided *t*-test.

**Table 1:** Patient characteristics and TCRb CDR3 sequencing metrics (staging is based on the 6th TNM edition). The maximum tumour diameter was measured on fresh specimens dissected through the long axis

Patient	Pathological characteristics and prior therapy	Region	Location within the tumour	Input DNA (ng)	Total TCRb reads	Productive TCRb reads	Unique TCRb reads	Highest frequency clone (%)
RMH002 Age 63 years, male	Stage T3N0M1, max. diameter 10 cm, grade 2–4, Sunitinib-treated	R2	Superior pole	2 000	3 069 283	2 615 838	14 563	2.02
		R6	Centre	2 000	1 334 098	1 183 323	15 730	2.58
RMH004 Age 61 years, male	Stage T4N0M1, max. diameter 14 cm, grade 1–4, treatment-naive	R2	Lateral pole	1 600	1 995 175	1 736 371	6 669	3.54
		R5	Inferior pole	1 290	2 150 919	1 899 305	16 289	1.65
		R7	Infero-medial	2 000	1 778 472	1 486 505	3 663	3.90
		R8	Posterior pole	2 000	2 080 796	1 818 886	3 035	3.19
EV003 Age 64 years, female	Stage T3N0M1, max. diameter 8 cm, grade 1–4, Everolimus-treated	R1	Centre	1 676	492 104	406 374	569	3.81
		R3	Medial pole	2 000	702 016	618 863	953	1.01
		R6	Superior pole	2 000	1 042 005	891 378	6 717	7.07
		R10	Lateral Pole	2 000	606 868	513 132	1 385	1.66
EV004 Age 57 years, female	Stage T2N0M1, Max. diameter 10 cm, grade 1–2, Everolimus-treated	R2	Infero-lateral	2 000	243 119	199 725	1 237	2.89
		R3	Infero- medial	471	910 205	838 404	1 752	10.61
		R4	Medial pole	1 873	460 035	372 847	367	4.22
		R6	Anterior pole	1 829	433 774	327 218	719	22.83

Paraffin-embedded tissue sections of different regions from all four cases were investigated by multi-immunoenzymatic staining, following protocols previously described 22,23. The antibodies used in this study were anti-human mouse monoclonals and included: FoxP3 (clone 236A/E7; Abcam, Cambridge, UK); CD4 (clone 4B12; Leica Microsystems, Newcastle upon Tyne, UK); and CD8 (clone C8/144B; Dako, Ely, UK). Scanned slide images were obtained with use of NanoZoomer Digital Pathology System (Hamamatsu, Japan) and individual T cell populations were quantified with use of ImageJ software on 10 representative areas/tumour section at ×40 magnification. Counting of positive cells was performed in a blinded manner by AF, TM and SAQ. Representative images of each section were acquired on a Nikon Eclipse E400 microscope equipped with ×10/0.30 and ×40/0.75 Plan Fluor objective lenses, using a Nikon DS-5Mc digital camera (all Nikon, Tokyo, Japan) and Adobe Photoshop CS4 image processing software (Adobe, San Jose, CA, USA).

## Results

DNA was extracted from three or four different tumour regions from each of the four primary ccRCCs. One of these tumours was treatment-naïve (RMH004), one had been treated for 16 weeks before surgery with the anti-angiogenic drug sunitinib (RMH002) and two had been treated with the mammalian target of rapamycin (mTOR) inhibitor everolimus for 6 weeks before the tumours were removed by surgery (EV003 and EV004) (for clinical and pathological characteristics, see Table 1). The CDR3 region of the TCRb was deep-sequenced in all samples; however, two attempts to sequence R8 from RMH002 failed and the sample was excluded from the analysis, leaving only two samples from RMH002 for analysis. All other regions were successfully sequenced, demonstrating that ultra-deep TCR sequencing allows the analysis of the T cell repertoire directly from DNA extracted from ccRCC samples without prior separation of the T cell population. Although the quantity of sequenced DNA was similar for most samples and they were all sequenced with an identical protocol, significantly fewer total reads were obtained from tumour samples from patients treated with everolimus [average total reads of everolimus-treated samples EV003 and EV004, 611 266 reads (SD 263 835); average total reads of samples RMH002 and RMH004, 2 068 124 reads (SD 572 250); *t*-test, *p <* 0.001] (Table 1). This suggests that the total number of rearranged TCRb, and thus the number of T cells in samples from tumours treated with everolimus, was significantly lower than in the other tumours. Sample EV004 R3 had a lower input DNA amount but contributed a higher number of total reads then other samples from the same tumour. Thus, the low number of T cells found in everolimus-treated samples did not appear to result from the inclusion of this sample with low DNA quantity.

**Figure 1:**
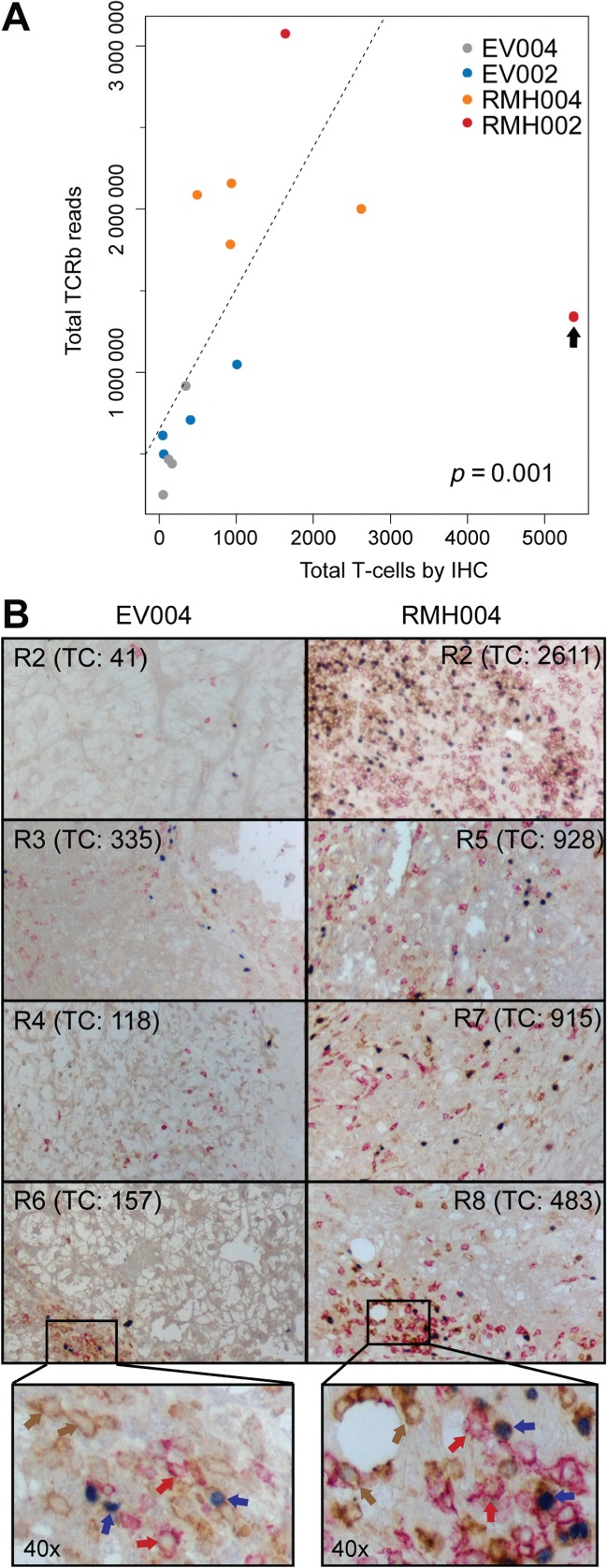
(A) Correlation of the immunohistochemically determined number of infiltrating T cells on the *x* axis with the total TCRb reads per sample on the *y* axis. The correlation is statistically significant based on the Spearman rank test and the *p* value is shown. A linear regression line was fitted through the data points after exclusion of the outlier R6 RMH002 (highlighted by an arrow); IHC, immunohistochemistry. (B) Representative results of the three-colour immunohistochemistry staining acquired with a ×10 objective; coloured arrows in high-magnification (×40) images highlight examples of T cells stained with the CD4 (brown), CD8 (red) and FOXP3 (blue) markers. The number of T cells (TC) counted in 10 different areas which are representative for each region are shown in brackets.

In order to investigate the ability of ultra-deep TCR sequencing to quantify intratumoural T cells, we stained formalin-fixed and paraffin-embedded (FFPE) sections obtained from tumour tissue adjacent to the fresh-frozen samples for the T cell markers CD4 (characteristic of T helper cells), CD8 (characteristic of cytotoxic T cells) and Foxp3 (characteristic of regulatory T cells). Infiltration by individual T cell populations was evaluated by a pathologist who was blinded to the sequencing results (Table 2). Total T cell infiltrates by immunohistochemistry showed a statistically significant correlation (Spearman rank test, *p =* 0.001) with the number of total TCRb reads (Figure 1A). R6 from RMH002 was an outlier, with > 5000 T cells detected by immunohistochemistry, the highest number of T cells across all samples, but only ∼1.3 × 10^6^ total TCRb reads. This may be the result of spatial heterogeneity of T cell infiltrates and random sampling effects, which could have led to an over-representation of T cells in the FFPE section compared to the adjacent issue block that was used for DNA extraction. Importantly, this analysis also confirmed lower numbers of intratumoural T cells in specimens from everolimus-treated patients compared to those from untreated or sunitinib-treated patients (Figure 1B). All major T cell subtypes (CD4^+^ T helper cells, CD8^+^ cytotoxic T cells and FOXP3^+^ regulatory T cells) were reduced in the everolimus-treated specimens (Table 2).

**Table 2:** T cell infiltrate per tumour region based on immunohistochemistry. T cell subtypes are shown as percentages of the total count

		Total lymphocyte count	CD8^+^	CD4^+^	CD4^+^FoxP3^+^
	(%)	(%)	(%)
RMH002	R2	1628	34.2	58.0	7.7
	R6	5357	52.5	41.6	5.8
RMH004	R2	2611	38.4	52.5	9.1
	R5	928	43.4	40.1	16.5
	R7	915	37.7	48.2	14.1
	R8	483	58.8	23.4	17.8
EV003	R1	49	53.1	46.9	0.0
	R3	396	27.3	68.9	3.8
	R6	1000	26.9	61.3	11.8
	R10	36	30.6	66.7	2.8
EV004	R2	41	48.8	46.3	4.9
	R3	335	67.2	30.4	2.4
	R4	118	30.5	63.6	5.9
	R6	157	22.9	73.9	3.2

**Figure 2:**
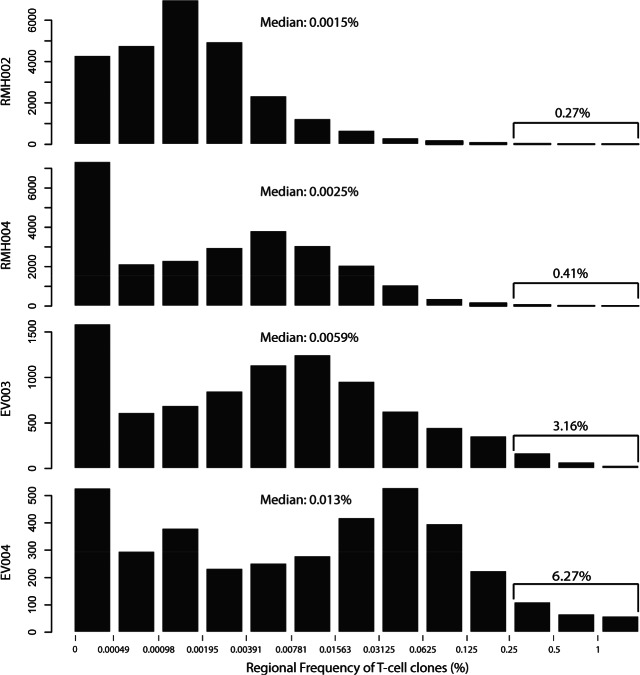
Histogram of T cell clone frequencies. The bin boundaries shown on the *x* axis represent the maximum regional frequency of the T cell clones in percent and are on a log 2 scale; the *y* axis shows the number of T cell clones in each frequency bin. The median percentage of all T cell clones/tumour and the percentage of T cell clones with a frequency of 0.25% and above are shown for each tumour.

Of the TCR sequences, 8–25% from each sample contained stop codons or frameshifts in the CDR3 region. These are unlikely to be functional and were removed. Only the remaining ‘productive TCRs sequences’ were used in further analyses. The total number of detected unique productive TCR sequences (each of them defining a unique T cell clone) per tumour region was between 367 and 16 289 (Table 1), indicating that intratumoural T cell populations were highly polyclonal in these ccRCCs. The median frequency of all individual T cell clones detected/tumour was in the range 0.0015–0.013%, demonstrating that the vast majority of T cell clones were present at low frequencies in each tumour (Figure 2). Only 0.27–6.27% of T cell clones in a single tumour were expanded to at least 0.25% of the T cell population in at least one tumour region (Figure 2). The maximum frequency of an individual T cell clone in a tumour region was 22.84% (detected in R6 of EV004) and in the range 2.58–7.07% in all other tumours (Table 1). These data would be consistent with a scenario where only a small number of T cell clones in each tumour expands to higher frequencies. Whilst this technology does not distinguish tumour-reactive T cells, the expansion of particular T cells clones could potentially be driven by their cognate antigen. In contrast, the majority of T cell clones, present at low frequencies, may be either low-affinity clones incapable of expanding in response to antigen or non-tumour reactive bystander lymphocytes.

Our next aim was to compare the TCR repertoire across multiple regions from each tumour to assess whether T cell populations in ccRCCs are spatially heterogeneous. Sequences which were detected in all regions from an individual tumour were classified as ubiquitous and all other T cell clones as heterogeneous. Only 0.24–16.82% of all T cell clones found in an individual tumour were detected ubiquitously in all regions (Table 3), whereas the majority of T cell clones were heterogeneous and, within the limits of our assay, spatially separated. Each sample had been sequenced to a depth in excess of several hundred thousand reads to allow the detection of low-frequency T cell clones (Table 1). However, the human T cell repertoire can be highly complex. For example, over one million unique TCRb sequences have been detected in the peripheral blood of an individual 24 and the theoretical number of different TCRb sequences which are expected to circulate in the blood of an adult is around 10 million 13. Thus, the number of unique T cell clones within a sequenced sample could be higher than the sequencing depth used in our study. This could lead to the failure to detect low-frequency T cell clones which may be present in a sample through random sampling effects, as previously described 21, and would result in an overestimation of intratumoural TCR repertoire heterogeneity.

**Table 3:** Intratumour heterogeneity of T cell clones

	Analysis based on all detected T cell clones/tumour				Analysis based on the 100 most frequent T cell clones/tumour			
RMH002	RMH004	EV003	EV004	RMH002	RMH004	EV003	EV004
Unique T cell clones	25 930	25 471	8809	3780		N/A		
Proportion of ubiquitous clones (%)	16.82	1.31	0.45	0.24	97.00	67.00	16.00	7.00
Proportion of heterogeneous clones (%)	83.18	98.69	99.55	99.76	3.00	33.00	84.00	93.00

**Figure 3:**
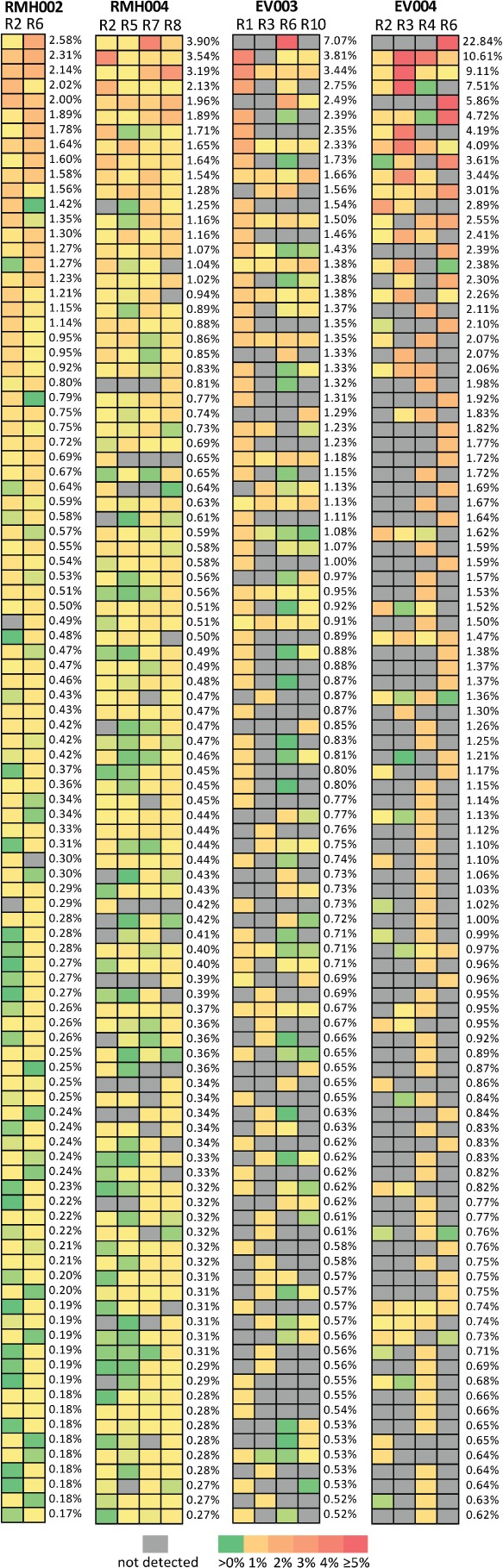
Heat maps showing the regional frequencies of the 100 most abundant T cell clones. The highest regional frequency/tumour is reported.

**Figure 4:**
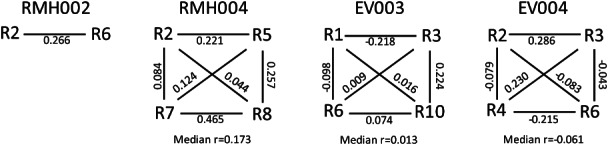
Pair-wise Pearson correlation coefficients based on the regional frequencies of the 100 most abundant T cell clones in each tumour.

Therefore, in order to attempt to avoid the potential impact of low-frequency clone sampling bias, we re-assessed heterogeneity, including only the 100 T cell clones found with the highest regional frequency across each of the four tumours; 3–93% of the 100 most abundant T cell clones in each tumour were still not ubiquitously detectable across all regions (Table 3). A heat map of the regional abundance of these T cell clones also demonstrated that the T cell populations found in ccRCCs are spatially heterogeneous and that different clones can be dominant in different tumour regions (Figure 3). Consistently, a Pearson correlation coefficient analysis of the abundance of individual T cell clones in any two regions per tumour revealed a poor correlation (Figure 4) for most pairs. Each of the 100 most abundant T cell clones per tumour was detected in > 2000 sequencing reads or 1.7% of all reads in at least one region (for detailed TCRb-sequences and read counts, see supplementary material, Table S1). Previous reproducibility and quality control experiments of TCRb sequencing demonstrated that the quantification of T cell clones with similar abundances are highly reproducible in repeat sequencing experiments from the same sample 21. Thus, the observed differences in regional frequencies within tumours are unlikely to be the result of detection and quantification biases but are likely to represent true differences between tumour regions. Comparison with clinical and pathological characteristics (Table 1) further demonstrated that heterogeneity was highest in the tumours pretreated for 6 weeks with everolimus, with 84% (EV003) and 93% (EV004) of T cell clones not detectable across all four regions. In comparison, only 33% of T cell clones were not detectable across all four regions from RMH004 and 3% of T cell clones in RMH002 were not detectable across the two sequenced regions. Data were only available from two regions in RMH002, which may explain the limited diversity compared to all other tumours from which four regions had been analysed.

Finally, we investigated whether we could identify any public TCRb sequences among the top 100 T cell clones in two or more patients. This did not identify any shared TCRb nucleotide or amino acid sequences between any of the four patients (data not shown).

## Discussion

This study shows that the T cell repertoire present in ccRCC tumour samples can be analysed directly by ultra-deep TCRb sequencing technology in DNA extracted from unfractionated specimens. Applied to multiple regions from each of four primary ccRCCs, this demonstrated that intratumoural T cell populations can be highly polyclonal, consistent with previous reports relying on a PCR-based CDR3 length analysis 25. Correlation analysis of TCRb reads and T cell infiltrates on histological sections validated the ability of ultra-deep TCRb sequencing to measure the abundance of T cells in these specimens. It is conceivable that sequencing based on quantification of intra-tumoural T cells may be more accurate than immunohistochemical methods, as the latter only survey a small section of the tumour, whereas DNA for sequencing analysis was extracted from larger, three-dimensional specimens. This may explain the outlier in Figure 1A, where IHC score and TCR reads show an imperfect correlation.

A number of potentially interesting and important observations can be made from our data, although, given the relatively small number of tumours examined, this is largely hypothesis-generating and requires further confirmation. Several T cell clones/tumour were present at frequencies suggesting clonal expansion, potentially as a response to activation by their cognate antigen. However, the vast majority of individual T cell clones were present at very low frequencies, suggesting that these clones have not undergone clonal expansion. These clones may not be able to recognize local tumour antigens or the clones may have been suppressed from expansion by virtue of local microenvironmental signals. Alternatively, many of these T cells may be present in the tumour sample because they are circulating through the tumour with the perfusing blood. Careful functional studies and comparisons with the T cell repertoire in the peripheral blood and normal kidney will be necessary to shed further light on this.

Restricting the analysis to the 100 most abundant regional clones/tumour displayed a heterogeneous distribution of individual T cell clones across different tumour regions. The variability in the frequency of individual T cell clones between tumour regions indicates that these clones are most likely infiltrating the tumour tissue and not simply contamination from the perfusing blood. T cells that are present at high levels in the perfusing blood would be expected to be detectable ubiquitously and at similar frequencies across different tumour regions. Exome sequencing of multiple tumour regions from individual ccRCCs recently demonstrated extensive genetic intratumour heterogeneity in these tumours 17,18. Importantly, heterogeneous subclonal mutations were spatially separated into different tumour regions. Thus, the identification of heterogeneous T cell populations in different tumour regions may indicate that T cell immune responses could be directed at spatially heterogeneous tumour neo-antigens present in distinct regions of these tumours. This demonstrates that multiple biopsies may be required to obtain a comprehensive overview of the response of the immune system to an individual cancer.

Comparison of the 100 most abundant TCRb sequences across the four cases did not identify any TCRb nucleotide or amino acid sequences which were shared by different patients. However, the question of whether common antigens are relevant in ccRCCs and can be recognized by T cells with such ‘public’ TCRb sequences, which arise independently in different patients, needs to be assessed in individuals which are matched for their HLA background, which was not the case in our study.

Our observation of a decreased polyclonality, coupled with an increase in the regional heterogeneity of T cell clones in the small number of tumours pretreated with the immunosuppressive mammalian target of rapamycin (mTOR) inhibitor everolimus, compared to the tumours which were untreated or treated with the anti-angiogenic drug sunitinib, may indicate that specific cancer therapies can influence the T cell repertoire and its distribution in solid tumours. Further analyses could be used to identify treatments that accentuate the number and abundance of T cell clones, to assess whether this may be associated with an increase in the efficacy of combination therapy with immune-stimulatory agents.

Our and others’ 16 data demonstrate that ultra-deep TCR sequencing can determine the clonality of T cells directly in DNA extracted from tumour tissues. Large collections of such samples are readily available for many tumour types, and these could be used to compare somatic genetic alterations with the T cell population present in the same sample. This may provide major insights into the parallel evolution and interplay of cancer genetics and the adaptive cellular immune response. For example, this may allow the investigation of immune editing processes, where cancer clones harbouring antigenic mutations may be lost due to eradication by T cells 26, or the analysis of differences in the immune response between poorly and highly immunogenic tumours, such as melanoma or renal cancer. This technology may also be useful to investigate clonality patterns of immune cell infiltrates, which may indicate a high probability of responding to immunotherapy, and the comparison of T cell repertoires of responders and non-responders may reveal how some cancers evade immunotherapy. Complementary approaches incorporating TCR sequencing, functional studies of T cells extracted from cancer specimens and multicolour immunohistochemistry to analyse different tumour regions may inform therapeutic decisions in the clinic, as these will provide both an understanding of the breadth and quality of the immune response in RCC.

In conclusion, we show that the clonality and abundance of intratumoural T cell populations can be analysed directly from DNA extracted from unfractionated ccRCC specimens. This provides a powerful new tool to investigate the magnitude and diversity of T cell responses in these tumours as potential biomarkers for prognosis and responses to novel immunotherapeutic agents. Similar to the recent identification of genetic and functional intratumour heterogeneity in ccRCC, T cell populations varied between different regions of the same ccRCC, demonstrating intratumour heterogeneity of T cell immunity. Thus, multiple biopsy approaches may be required to fully characterize the immune response to an individual tumour.

## Supplementary Material on the Internet

The following supplementary material may be found in the online version of this article

Table S1 Read counts across individual tumour regions for the 100 TCRb DNA sequences found, with the highest regional frequency in each of the four tumours
